# Perspectives of Psychotherapists and Psychiatrists on Mental Health Care Integration Within Primary Care Via Video Consultations: Qualitative Preimplementation Study

**DOI:** 10.2196/17569

**Published:** 2020-06-18

**Authors:** Mariell Hoffmann, Michel Wensing, Frank Peters-Klimm, Joachim Szecsenyi, Mechthild Hartmann, Hans-Christoph Friederich, Markus W Haun

**Affiliations:** 1 Department of General Internal Medicine and Psychosomatics Heidelberg University Heidelberg Germany; 2 Department of General Practice and Health Services Research Heidelberg University Heidelberg Germany

**Keywords:** video consultations, videoconferencing, telehealth, integrated care, mental health, health services research, qualitative study, preimplementation

## Abstract

**Background:**

Many patients with mental disorders remain untreated. Video-based mental health care demonstrates comparable effectiveness to face-to-face treatments and is a promising mode for delivering specialized care within primary care. Nevertheless, professionals struggle with implementing video consultations in their daily practice. Specifically, little is known about mental health specialists’ acceptance of mental health video consultations in routine practice. The PROVIDE (ImPROving cross-sectoral collaboration between primary and psychosocial care: An implementation study on VIDEo consultations) project aims to improve cross-sectoral collaboration between primary and psychosocial care through implementing video consultations in primary care. To increase the uptake of video consultations, it is crucial to account for necessary prerequisites and to tailor interventions to the needs of the target group prior to implementation.

**Objective:**

The aim of this study was to explore the acceptance of video consultations embedded in primary care from the perspectives of mental health specialists in Germany.

**Methods:**

We conducted a qualitative, exploratory, preimplementation study in urban and rural counties. We conducted three semistructured focus groups with 11 mental health specialists. We used qualitative content analysis combining an inductive-deductive approach, applying the Tailored Implementation in Chronic Diseases (TICD) framework to the text material, which comprises individual health professional factors; patient factors; professional interactions; incentives and resources; capacity for organizational change; social, political, and legal factors; and guideline factors.

**Results:**

Against the background of long waiting times and a shortage of mental health specialists, especially in rural areas, participants valued video consultations as a potential means to improve access to mental health care. With respect to the TICD framework domains, the participants most often discussed individual health professional factors, followed by patient factors. All participants highlighted the importance of a trusting relationship between the patient and the therapist and doubted whether such a relationship could be established through video consultations (11/11, 100%). However, participants considered mental health specialist video consultations to be particularly suited for patients in rural areas, those with impaired mobility, and those who may otherwise remain untreated (6/11, 55%). Most participants expected video consultations to help the aforementioned patient groups avoid tedious searching for an available therapist and save on travel time and, therefore, improve access to specialized care for patients (7/11, 64%). Moreover, the participants expected video consultations to improve collaboration with the family physician (6/11, 55%). Finally, participants identified organizational aspects, such as reliable scheduling, the duration of the individual consultation (9/11, 82%), and reimbursement conditions (7/11, 67%), as key drivers for the acceptance and adoption of the model.

**Conclusions:**

While mental health specialists expect video consultations to improve access to specialized care for some patients, they consistently wonder whether such consultations can establish a trusting patient-therapist relationship. When implementing video consultations, these concerns should be addressed by training providers in managing technology-based treatment settings, with extra consideration for fostering the patients’ and therapists’ engagement.

**Trial Registration:**

German Clinical Trials Register DRKS00012487; https://tinyurl.com/uhg2one

## Introduction

### Background

Many patients with common mental health conditions, such as depression, anxiety, or somatic symptom disorders, solely consult their family physicians. When seeking specialized mental health care, patients face the challenge of limited access, especially in rural areas. A significant number of these patients remain undiagnosed or receive psychiatric medication prescriptions, although most would prefer psychological treatment [1,2]. According to the American Psychiatric Association and the Academy of Psychosomatic Medicine, mental health care specialistss—namely, psychotherapists and psychiatrists—are required to provide patient-centered care and improve access of care (eg, by supporting integrated delivery and providing multiple points of access) [3]. Internet-based integrated care models such as video consultations are one way to address the demands for mental health care, provided that family physicians and mental health specialists can overcome system barriers and foster cross-sectoral communication. Several studies demonstrated acceptable feasibility and equal effectiveness of video consultations compared to face-to-face treatments in psychotherapy [4-10]. Additionally, video consultations can save costs and travel time [4,11]. Patients generally accept video consultations and are often satisfied with this mode of treatment delivery [6,8,12]. However, regarding the uptake of best evidence in practice, one consistent research finding is the gap between evidence-based interventions and their adoption in clinical practice. To bridge this gap, it is crucial to identify user acceptance of interventions and to explore prerequisites among the target group of intervention users to tailor the intervention to their needs and foster the adoption of best practice standards into routine care [13]. Several projects evaluated video-based integrated care models, but little is known regarding how mental health specialists, especially in Germany, perceive the acceptance of real-time video consultations between patients presenting to the primary care practice and off-site mental health specialists.

### Mental Health Specialist Video Consultations Embedded in Primary Care

The PROVIDE (ImPROving cross-sectoral collaboration between primary and psychosocial care: An implementation study on VIDEo consultations) project aims to improve cross-sectoral collaboration between primary and psychosocial care through implementing video consultations in primary care. The intervention involves clinical diagnostics, care planning, and crisis management or brief psychotherapy and is limited to a maximum of five consultations. Specifically, mental health specialists will conduct video consultations with patients with depression and/or anxiety who are under the care of his or her primary care family physician. The PROVIDE project consists of three stages: first, we identified barriers and facilitators of stakeholders to determine their needs and tailor the model accordingly (preimplementation phase: PROVIDE-A). Supplementing our recently published work on the perceptions of family physicians regarding mental health specialist video consultations in primary care [14], this paper presents the results for psychotherapists and psychiatrists from the initial preimplementation phase of the PROVIDE project. Second, the video consultation model will be put into practice and evaluated regarding the acceptance and practicability within a pilot study (feasibility study: PROVIDE-B) [15]. Third, the model will be optimized where necessary and tested in a randomized controlled trial (RCT) with respect to the extent to which the burden of depression and anxiety of patients changes, as well as patient outcomes, as compared to patients who received usual care (implementation study, RCT: PROVIDE-C).

### Purpose of the Study

This qualitative study from the preimplementation phase of PROVIDE-A aimed to (1) identify perceived acceptance of the intervention among mental health specialists and (2) shed light on prerequisites—namely, barriers and facilitators—for the implementation of mental health specialist video consultations.

## Methods

### Study Design

In order to assess the acceptance of mental health specialist video consultations, we conducted a cross-sectional, qualitative, exploratory preimplementation study with mental health specialists. The results of this study will be used to further tailor the intervention model to the practical needs of mental health specialists and will then be evaluated within a following feasibility study [15]. We conducted three focus groups with a total of 11 participants. By using focus groups, we were able to collect diverse perceptions and experiences, which focus group participants typically disclose to each other and on which they collectively reflect [16]. This study was approved by the Ethics Committee of Heidelberg Medical School (Reference S-197/2017) and was preregistered with the German Clinical Trials Register (DRKS00012487). We followed the COREQ (COnsolidated criteria for REporting Qualitative research) guidelines for reporting study results (see Multimedia Appendix 1) [17]. The authors had full access to all the data in this study and take complete responsibility for the integrity of the data and the accuracy of the data analysis.

### Participants and Recruitment

We used purposeful sampling to obtain a broad range of perceptions [18]. To be eligible, mental health specialists had to be registered with the Association of Statutory Health Insurance Physicians in one of the five counties covered in this study; the five counties were selected from a total of 35 counties in Baden-Wuerttemberg, one of 16 German federal states. Overall, 583 mental health specialists were eligible. We chose a stratified randomized sample of two waves—first wave in June 2017 (N=100); second wave in September 2017 (N=195)—in order to recruit at least 10 participants for the focus groups. Due to the shortage of mental health specialists, particularly in rural areas, we aimed to explore potential distinctions between *rural and suburban* areas and *urban* areas. Therefore, we used the Degree of Urbanisation (DEGURBA) classification from the European Commission to distinguish the urban and rural counties [19]. Overall, 275 mental health specialists from cities, 261 mental health specialists from suburbs, and 47 mental health specialists from rural areas were eligible. We sent a written questionnaire to a total of 295 stratified randomized mental health specialists regarding their perception of mental health specialist video consultations (results not presented here) and invited them to participate in a focus group. If no response had been received within 3 weeks, we reminded all nonresponders via follow-up telephone calls (up to three calls). To schedule the focus groups, we contacted all 27 eligible mental health specialists, by telephone, who had expressed interest in participating. A total of 15 mental health specialists subsequently declined study participation, mainly due to time restrictions (9/15, 60%). One participant agreed to participate in a telephonic interview but is not included in the analysis, as we focus on collective reflections on the intervention. Overall, we conducted three semistructured focus groups with a total of 11 participants. We stopped data collection and started data analysis when no new insights occurred in the data and the content began to repeat. We offered a nonadvertised, individual monetary compensation of €50 (US $55) to all participants.

### Focus Groups

For the development of a questionnaire guide (see Multimedia Appendix 2), we followed established recommendations [20] and used a team-based approach—M Hoffmann, sociologist; MW, implementation science; M Hartmann, psychotherapist; and MWH, internal medicine specialist—advancing to specific open-ended, jargon-free questions from the initial formulation of key questions in a logical sequence. The interviews focused on (1) how mental health care providers perceive current mental health care, (2) the acceptance of and intent to adopt mental health specialist video consultations, and (3) factors that promote or inhibit the use of the model. Before conducting the focus groups, we piloted the guide on one mental health specialist and modified it accordingly. The question guide was also reviewed after the first focus group with mental health specialists. The first author (M Hoffmann: PhD student, master’s degree in sociology, and expertise in qualitative research) and the last author (MWH: MD, internal medicine specialist, senior researcher, and content expert for mental health services), who had no contact or relationship with any participant prior to the study, conducted the focus groups. The focus groups, which ranged from 2 to 5 participants and lasted 80-110 minutes, were conducted at Heidelberg University Hospital between August and November 2017; participants were from one urban and four rural or suburban counties. We used the question guide to encourage discussion and to collect comparable data through introducing similar discussion topics. To stimulate the discussion, the moderators presented a video clip illustrating the model using an example of a fictional patient suffering from depression. We made the study objectives and data protection guidelines transparent to all participants, who provided written consent prior to data collection. We guaranteed the absence of nonparticipants during the focus groups. We audio recorded all focus groups and uploaded them to a secure server based at Heidelberg University Hospital, which was only accessible to the research team. Additionally, we made field notes.

### Analysis

A professional transcription service completed verbatim audio transcriptions of all focus groups. We did not return transcripts to participants for comment. We pseudonymized all transcripts according to data protection guidelines. Two members of the research team (M Hoffmann and MWH) independently performed content analysis [21] using MAXQDA 2018, following a two-step, deductive-inductive, and data-driven development of a system of categories. First, we used the Tailored Implementation in Chronic Diseases (TICD) framework [22] as a coding framework to deductively identify relevant determinants, focusing on barriers and facilitators of the acceptance of mental health specialist video consultations. The TICD framework is regarded as particularly appropriate for processing data focusing on implementation factors in order to tailor an intervention to specific needs. Second, we inductively generated the subcodes based on the analysis of every transcript. Both researchers compared their analysis, and disagreements throughout the process were discussed and resolved among the research team. We analyzed the remaining transcripts based on the developed coding system. In order to ensure that all key aspects were represented in the coding system, codes were consequently modified when new aspects emerged. Table MA3-1 in Multimedia Appendix 3 provides a summary of the coding system. We did not ask participants to provide feedback on the findings.

## Results

### Overview

Table 1 shows the characteristics of the 11 participants. Specifically, we interviewed 4 licensed psychological psychotherapists (36%), 3 physicians with board certification in psychosomatic medicine and psychotherapy (27%), 3 board-certified psychiatrists (27%), and 1 medical doctor with an additional qualification in psychotherapy (psychotherapeutisch tätiger Arzt, in German) (9%).

### Depiction of Current Practice

Three main topics related to the current practice of patients with mental disorders in outpatient care emerged. First, long waiting times and an insufficient number of available mental health specialists (10/11, 91%) were the most frequently discussed issue, particularly due to the urban-rural divide. Especially for more severely affected patients, participants perceived long travel distances as well as long waiting times as barriers for the uptake of psychotherapy.

Sometimes the patients must wait for a very long time and sometimes they are also very burdened. They are also not able to call five other psychotherapists and drive to all of them.

Participant #3, focus group #2

Second, mental health specialists identified another health care gap regarding some patient groups and their difficulties in finding a therapist (6/11, 55%), such as the elderly, patients with impaired mobility, as well as severely affected patients (eg, those with borderline personality disorders). Third, beyond patient-related factors, mental health specialists perceived a lack of collaboration with family physicians (6/11, 55%), for instance, the absence of comprehensive referral letters (4/11, 36%) and the lack of an opportunity to consult with specialists in cases of diagnostic uncertainty (5/11, 45%). This was often associated with limited time resources of the mental health specialist and the family physician.

**Table 1:** Characteristics of the sample participants.

Characteristic		Value (N=11)
Sex (male), n (%)		2 (18)
Age in years (n=10), mean (SD)		52.3 (12.8)
Years in office-based practice (n=8), mean (SD)		13.1 (11.8)
**Areas of recruitment, n (%)**		
	Cities (ie, densely populated areas)	6 (55)
	Towns and suburbs (ie, intermediate-density areas) and rural areas (ie, thinly populated areas)	5 (45)
**Therapeutic approach, n (%)**		
	Psychodynamic psychotherapy	5 (45)
	Psychoanalysis	3 (27)
	Behavior therapy	3 (27)

### Factors Determining the Acceptance of Mental Health Specialist Video Consultations

We present barriers and facilitators regarding the acceptance of mental health specialist video consultations in a descriptive manner alongside the seven TICD framework domains in descending order by code frequency. Two domains—namely, individual health professional factors and patient factors—were identified as highly relevant and most frequently discussed. Some domains—namely, social, political, and legal factors as well as guideline factors—were hardly discussed and are not reported.

### Individual Health Professional Factors

Regarding the acceptance of the intervention model, mental health specialists most frequently discussed individual health professional factors (11/11, 100%). For instance, they perceived some organizational factors as potential challenges.

Of course, there are organizational issues which need to be addressed. Primarily, how can it be managed without any disadvantages for me...I still do not have more capacities this way.

Participant #1, focus group #2

According to most of the participants (9/11, 82%), time and duration of the individual video consultation may be one major challenge of implementing the intervention. For example, some participants perceived a video consultation to be more exhaustive compared to a face-to-face appointment and, therefore, considered 25 minutes to be an appropriate duration. Others emphasized that mental health specialist video consultations should last as long as face-to-face consultations, which usually run for 50 minutes. They argued that this would contribute to establishing a therapeutic setting of the video consultation and, therefore, 50 minutes would provide a sufficient duration to get a comprehensive understanding of the patients’ situations. When asked about potential benefits or expected outcomes, participants commonly highlighted the collaboration with the family physician (6/11, 55%) and help for patients (7/11, 64%). Overall, mental health specialists expected fewer advantages for themselves than for patients, especially compared to their everyday practice in the face-to-face setting. As a major issue, which has been subject to every focus group, mental health specialists questioned whether video consultations can be an appropriate and suitable means to deliver specialized mental health care to patients in need (11/11, 100%).

So, the exclusive situation, precisely that you do not evade...this gets somewhat socially lost as a fundamental, therapeutic experience.

Participant #3, focus group #3

Mental health specialists most commonly highlighted the advantages of the traditional face-to-face setting, while emphasizing some disadvantages of mental health specialist video consultations. Specifically, they underscored a lack of personal interactions and nonverbal cues, which may impede the therapeutic relationship and engagement (8/11, 73%).

This may also entail several disadvantages for me. Because it is a different setting, sitting together in a room...I believe the ritual of doing therapy together in a room is too important.

Participant #1, focus group #2

Some participants considered the model to work better when the patients already had previous appointments with the therapist and when a sustainable patient-therapist relationship already existed (3/11, 27%). Others (4/11, 36%) expected the model not to work as therapy in the traditional sense. Moreover, they emphasized that mental health specialist video consultations might dehumanize the therapist-patient relationship and, consequently, should not replace face-to-face therapies. Against this background, mental health specialists considered it to be essential to clearly define the purpose of the intervention and how it can make a significant contribution to improve the patients’ situations.

I believe it is important to define the framework. What is supposed to happen? Psychotherapeutic video consultation implies the message that something therapeutic happens. But we all shared to some extent the opinion that it is not likely that something psychotherapeutic can really happen.

Participant #3, focus group #2

From the perspective of the mental health specialists, the model may be particularly suited for an initial mental health consultation. Within this setting, mental health specialists could serve as experts to provide more rapid access to mental health care. Some participants stated that their professional and therapeutic habits, as well as their daily routines to work in a face-to-face setting, might lead to a more conservative attitude toward digital health interventions, in general, and mental health specialist video consultations, in particular (5/11, 45%). One mental health specialist stated the following:

And getting used to this, yes. That is certainly a difference. Now with the monitor and talking to the patient in this way. The familiarization. But I think it is also exciting.

Participant #1, focus group #1

Using the intervention in practice could reduce reservations and enable mental health specialists to become familiar with the video consultation.

### Patient Factors

First, within this domain, mental health specialists most frequently discussed appropriate target groups for the intervention (10/11, 91%). They considered mental health specialist video consultations to be most suitable for patients with impaired mobility (6/11, 55%) who may otherwise remain untreated. The following statement by another participant emphasized that the model could serve as an alternative model to deliver mental health care, especially in rural areas.

...this is not supposed to replace conventional psychotherapy; rather, just for particular cases, right? For people who just live too far away, where the infrastructure is very, very poor.

Participant #1, focus group #1

In contrast, some participants mentioned patients with specific mental health conditions, such as anxiety disorders, or patients with posttraumatic stress disorders (3/11, 27%) as potentially less suitable for mental health specialist video consultations. Hence, they stated that it should be clearly specified which patients are the main target group to tailor the intervention to their needs. Furthermore, some mental health specialists indicated that in the event of an emergency, it should be clearly defined who is on site in general practice and responsible for the patient (3/11, 27%). Second, regarding patient barriers, participants anticipated that some patients might have concerns about the video technology, as such (5/11, 45%). This might lead to less interest or refusal to conduct mental health specialist video consultations, especially regarding the elderly. Therefore, it might take some extra effort to encourage the acceptance of mental health specialist video consultations. Considering potential benefits for patients, they valued quicker help, low-threshold access, and the initiation of psychotherapy as main facilitators.

I could very well imagine doing something like this. To offer a low-threshold service, to initiate psychotherapy, very carefully and sensitively.

Participant #4, focus group #2

As a potential result, long waiting times, a tedious search for an appointment, as well as long travel distances could be avoided.

### Professional Interactions

Against the background of a lack of collaborative exchange with family physicians as outlined above, mental health specialists expected professional interactions with the family physician to be a potential benefit from, and facilitator for, mental health specialist video consultations (7/11, 64%). Some participants considered the model to foster collaboration with the family physician and highly valued the possibility to give feedback to the family physician and better involve him or her in the process of care (5/11, 45%). Specifically, they considered brief case discussions or the virtual handing over of the patient by the physician as advantageous.

For the initial contact, it would certainly be nice if the general practitioner would facilitate it by introducing it [the model] and state what he has thought of. To already sort of point me in the right direction...And then I could, I could go deeper with the patient. And maybe at the end, I could share the result, with him [family physician]...What, from my view, I believe could, should be done.

Participant #1, focus group #2

Some participants also discussed the possibility of a consultation not only between the patient and the mental health specialist but also with the family physician, who could attend the video consultation (3/11, 27%). In this setting, mental health specialists would want to discuss the patient’s condition with the patient and the family physician. As a result, this could foster collaboration between the family physician and the mental health specialist, as well as the patient’s involvement.

I think of a trialogue, for example, between the general practitioner, the patient, and psychotherapist and one asks the psychotherapist for his suggestion.

Participant #4, focus group #3

Yes, I also find the trialogue very exciting, because this way the patient and the general practitioner are on a triangulating level with me as a consultant. This also changes the relationship between them and adds something new.

Participant #1, focus group #3

Furthermore, within this domain, some mental health specialists discussed the distribution of roles and responsibilities (4/11, 36%). First, regarding family physicians’ roles, referring patients would be an essential task. Also, as described above, participants considered as potentially helpful the introduction of the patient to the mental health specialist at the beginning of the video consultation. Second, mental health specialists regarded themselves as expert consultants, especially against the background of video consultations as a different model of delivery compared to traditional face-to-face psychotherapy.

And this would possibly also be the framework; for the patient, the psychotherapist would be the expert, so to say...Sort of, consulting someone and to get clarification in a particular, temporarily defined setting, and where the patient could possibly save trips.

Participant #3, focus group #3

### Incentives and Resources

First, within this domain, the majority of participants extensively discussed costs and payment conditions related to the intervention (7/11, 64%). As outlined in the first domain, participants do see more disadvantages than benefits, especially compared to their daily face-to-face working practice. Therefore, mental health specialists regarded a prerequisite of a reasonable financial remuneration to be an incentive to conducting mental health specialist video consultations themselves.

I believe it is important to settle financial issues. Particularly, because therapists are not at all dependent on this [the model].

Participant #1, focus group #1

Some participants stated that the model should include remuneration of video consultations to be equal to face-to-face sessions (3/11, 27%) or higher (2/11, 18%), in order to enhance the mental health specialists’ motivation to conduct video consultations. Also, considering financial compensation, 2 participants (18%) stated that the video consultation sessions should be remunerated even when patients were not able to keep the appointment. Otherwise, this might negatively affect mental health specialists’ acceptance of the treatment model, as mental health specialists in Germany receive a payment in cases where the patient misses an appointment because most mental health specialists run an appointment-based practice. In addition to that, they underscored stable network connectivity, high visual definition, minimized speech delay, minimized interruptions, and the preinstallation of equipment as essential. Technical equipment should be provided and not be paid for by the mental health specialists themselves. Considering the coordination of appointments, participants stated that fixed time slots are a prerequisite as well as the duty of primary care practices to efficiently implement the intervention into their daily working practice. Overall, participants required organizational demands to be settled in order to commit themselves to mental health specialist video consultations, as illustrated by the following quote:

If we do not consider all organizational issues, as how is it practically possible...if I look at it from a completely IDEALISTIC perspective, then this would not be a bad idea. Let´s say as an initial contact.

Participant #1, focus group #2

### Capacity for Organizational Change

Mental health specialists discussed aspects regarding the capacity for organizational change only when explicitly asked about their perceptions of the readiness for change of mental health specialists in general. Some participants expected heterogeneous attitudes among mental health specialists in general. Others expected younger mental health specialists to be more curious and open minded (2/11, 18%).

The people above 50 might be a little bit more reluctant; about the younger ones, I am not entirely sure. But I could imagine that they are more adventurous.

Participant #2, focus group #1

One participant suspected that either intrinsic motivation—namely, providing help for patients—or financial incentives would be a potential means to drive the capacity for organizational change.

I could imagine that those colleagues would agree to this [the model], who do this as additional benefit for the patient. Or one would have to compensate it in a way that it becomes financially profitable.

Participant #5, focus group #2

### Differences in Views in Relation to Urbanization and Therapeutic Orientation

To account for potential distinctions, we compared data from cities (6/11, 55%) to that of suburbs and rural areas (5/11, 45%), as well as different therapeutic orientations—namely, psychodynamic psychotherapy and psychoanalysis psychotherapy (8/11, 73%) as well as cognitive behavioral therapy (3/10, 27%). First, a comparison of the degree of urbanization revealed that participants from cities (4/6, 67%) more frequently mentioned the appointment time and scheduling of the mental health specialist video consultations and technical requirements as prerequisites compared to participants from less-urbanized areas. Also, mental health specialists from cities (4/6, 67%) more often highlighted the possibility to collaborate with the family physician compared to specialists from suburbs or rural areas (2/5, 40%). Second, regarding different therapeutic approaches, we found that cognitive behavioral therapy–oriented participants considered clearly defined responsibilities in the event of an emergency (3/3, 100%), whereas participants with other therapeutic orientations did not discuss this issue. Also, cognitive behavioral therapy–oriented participants more frequently considered technical aspects and an impersonal setting within the mental health specialist video consultations model as potential barriers for some patients (3/3, 100%), compared to specialists practicing psychodynamic psychotherapy and psychoanalysis psychotherapy (4/8, 50%). Taking into consideration that all participants underscored the importance of physical presence for psychotherapy, mental health specialists with a background in psychodynamic psychotherapy and psychoanalysis psychotherapy particularly mentioned patients with impaired mobility as an appropriate target group for the model (5/8, 63%). Cognitive behavioral therapy–oriented participants (1/3, 33%) indicated these patients as a target group less frequently.

## Discussion

### Principal Findings

The aim of this study was to explore how mental health specialists perceive the acceptance of mental health specialist video consultation embedded into primary care practice against the background of their perception of current mental health care. Most mental health specialists described the latter as insufficient due to long waiting times and a lack of therapeutic capacities, especially in rural areas. We explored crucial barriers and facilitators of mental health specialist video consultations as a potential mode to deliver mental health care. First and foremost, mental health specialists questioned whether a stable therapeutic alliance can be established, especially with respect to the therapeutic alliance and engagement. Therefore, they considered mental health specialist video consultations to be particularly suited for an initial consultation and diagnosis, rather than a genuine alternative to face-to-face psychotherapy. Second, regarding mental health specialists’ perceptions of the patient perspective, they highlighted fast access to mental health care, especially for patients with impaired mobility, as a major benefit of the model. Participants considered mental health treatment via video consultations not to be a barrier but to be rather predominantly advantageous for most patients. Third, apart from an intrinsic motivation—namely, to provide faster access to mental health care—mental health specialists considered financial incentives as instrumental. Fourth, participants underscored important organizational factors, such as stable network connectivity, high visual definition, and minimized interruptions. Therefore, training sessions in order to familiarize users with the video consultation in practice are key drivers for the intervention.

### Strengths and Limitations

Our study has some limitations. First, due to the small sample size and only one mental health specialist from rural areas participating in our study, the results and distinctions between participants from different areas are reliable to a limited degree. Second, none of the participants had conducted mental health specialist video consultations before participating in the study, since we conducted the focus groups prior to implementation of the proposed intervention model. It is plausible that some mental health specialists would revise their initial perceptions regarding the intervention model after the actual implementation. However, by assessing the perceptions before the implementation, we explored necessary prerequisites for further tailoring mental health specialist video consultations to the mental health specialists’ needs, which is key for the adoption of a new mode of delivering mental health care. Third, we did not apply a technology acceptance framework (eg, technology acceptance model [TAM] or unified theory of acceptance and use of technology [UTAUT]). Nevertheless, while such models support the explanation of the potential user's behavioral intention to engage with a technological innovation [23], our focus was on meso-level implementation in a broader sense. Thus, we employed the TICD framework for guiding both the analysis of our data and interpretation of our findings. Finally, not all barriers related to the proposed model may have been referred to by the study participants. For example, it is plausible that the distance between the patient’s home and the general practice itself could be a problem for patients struggling with limited mobility. However, we would argue that patients usually need to see their general practitioner eventually for regular checkups and/or the monitoring and treatment of medical diseases. From a patient-centered perspective, the proposed video consultations could be included in these appointments. At the very least, patients would save time and avoid travel related to additional in-person visits with mental health specialists.

### Comparison With Prior Work

Previous work on video consultations, and telemedicine in general, has mainly focused on efficiency trials, satisfaction, and attitudes after the implementation of video consultations. A large body of research points to a comparable effectiveness of video consultations and face-to-face consultations [4,24-27]. Referring to the patients’ perspective, many studies found high satisfaction rates with video consultations for addressing various health conditions [4,9,27,28]. In contrast, another study found higher satisfaction rates for patients than for clinical staff [27]. However, within this work (ie, focusing on chronic health conditions), reasons for the lower level of satisfaction among clinical staff remained unclear. Our results add that mental health specialists were most often concerned about a potential dehumanization of the therapeutic alliance caused by a lack of personal interactions and nonverbal cues. Another online survey study among stakeholders in eight European countries found that care providers had particular concerns regarding the therapeutic alliance [29], which is in line with our findings. However, we provide additional insights into the perspective of mental health specialists, as we also investigated necessary prerequisites and key drivers, such as the collaboration with the general practitioner, specific target groups, and the virtual handing over of the patient by the physician. A systematic review on the therapeutic alliance in videoconferencing found that therapists rated the therapeutic alliance as high, although not as high as their clients did, but often increasing over the course of therapy [30].

Our study, as well as other studies, point to the importance of training for mental health specialists regarding the use of video consultations [30-34]. Training sessions should impart knowledge regarding the preparation and technical conduct of video consultations as well as encourage the therapist’s ability for reflection. This might potentially reduce some reservations. Additionally, we found that training sessions should especially focus on establishing a therapeutic alliance via video, such as ways to greet the patient virtually and open the session, and on fostering therapeutic and patient engagement as crucial preconditions to adopt mental health specialist video consultations. We found that mental health specialists evaluated the acceptance of mental health specialist video consultations almost entirely in comparison to face-to-face consultations and particularly emphasized the benefits and qualities of the latter. Specifically, we found that participants in our study mainly did not consider mental health specialist video consultations as a favorable alternative mode to delivering mental health care, which some mental health specialists associated with their professional habits and daily routines. Exceptions were mental health specialist video consultations for otherwise untreated patients, especially in remote and rural areas, and mental health specialist video consultations as initial consultations. Besides training, previous studies also revealed that an existing relationship between the patient and the health care provider is crucial [35,36], which some mental health specialists in our study also indicated. When it comes to the acceptance and adoption of new innovations, such as mental health specialist video consultations, the perceived compatibility of innovation with the values and norms of the potential adopters are key elements [37]. Therefore, mental health care delivered via video consultations should focus on these target groups in order to increase the compatibility of mental health specialist video consultations with the norms of mental health specialists and their daily practices. Previous studies on this topic have been limited to perceptions after the treatment model has already been implemented. Regarding our previous work on family physicians’ perceptions of mental health specialist video consultations in primary care [14], this study adds necessary insights from the perspective of mental health specialists on the proposed treatment model. Comparable to the main barrier perceived by mental health specialists, which is the therapeutic alliance, family physicians in our previous study underscored a lack of personal interactions and nonverbal cues that may impede therapeutic engagement. Also, they valued benefits not only for patients but also for the family physicians themselves—namely, that mental health specialist video consultations may free up resources for the primary care practice. However, in this study, we found that mental health specialists perceived more disadvantages for themselves and their therapeutic work, as described above.

### Conclusions

To increase access to specialized care, emergent technologies like interactive video consultations, which are known to be highly accepted by patients, will probably complement traditional in-person consultations in future health care systems. However, we found that mental health specialists are still somewhat skeptical concerning the impact on the patient-therapist relationship. To foster the implementation and adoption of mental health specialist video consultations in primary care, it is essential to train providers in managing technology-based treatment settings with extra consideration for preserving therapeutic alliance and to familiarize mental health specialists with video consultations. Based on the findings from this study and our previous work, we started a feasibility study (trial registration number: DRKS00015812) to further tailor the intervention model to the participants’ needs and to evaluate the acceptance and practicability of mental health specialist video consultation in primary care [15].

## Appendix

Multimedia Appendix 1 COREQ (COnsolidated criteria for REporting Qualitative research) checklist.

Multimedia Appendix 2 Semistructured guide for focus groups and telephone interview.

Multimedia Appendix 3 Description of the coding system.

## Abbreviations

BMBF: Federal Ministry of Education and Research
COREQ: COnsolidated criteria for REporting Qualitative research
DEGURBA: Degree of Urbanisation
PROVIDE: ImPROving cross-sectoral collaboration between primary and psychosocial care: An implementation study on VIDEo consultations
RCT: randomized controlled trial
TAM: technology acceptance model
TICD: Tailored Implementation in Chronic Diseases
UTAUT: unified theory of acceptance and use of technology
