# Supplementary material for: Perspectives of Psychotherapists and Psychiatrists on Mental Health Care Integration Within Primary Care Via Video Consultations: Qualitative Preimplementation Study
Source: J Med Internet Res. 2020 Jun 18;22(6):e17569. doi: 10.2196/17569 (PMC7333073; doi:10.2196/17569)
Supplement: Multimedia Appendix 2 [file jmir_v22i6e17569_app2.docx]

**Multimedia Appendix 2**

**Semi-structured guide for focus groups and telephone interview**

*(Finalized version as of August 2017)*

*A. Current health care of patients with mental health conditions in primary care*

- Considering outpatient services, what do you think of the current health care of patients with mental health conditions?
  - To what extent do you perceive gaps in the current health care system?
  - If applicable, how do you personally tackle with these gaps?
- How would you describe the typical patient suffering from a mental health disorder in your practice?
  - If applicable, how would you describe any peculiarities entailed in health care delivery for patients with mental health conditions?
- How would characterize sound health care delivery for patients with mental health conditions?
- If applicable, do you benefit from a local network involving family physicians in outpatient care?

*B.* *Introduction of the PROVIDE intervention model, implementation potential und compatibility (e.g. readiness for change)*

- What do you think of the proposed treatment model in the PROVIDE project?
- To what extent do you expect to benefit from such a model in your personal working environment?
- Which patient groups seem suitable for receiving treatment under the proposed model?
  - What could be reasons that patients might not make use of the proposed video consultations?
- From your point of view, are mental health specialists currently willing to employ the proposed treatment model?

*C. Possible factors that promote or inhibit implementation of video consultations in primary care*

*(Determinants of practice)*

- Which eventual challenges and concerns do you see for
  - patients
  - health care personnel
    - in terms of time, location, organization and training
- Which benefits do you see for
  - patients
    - To what extent do you expect patients with mental health conditions to benefit from the treatment model?
  - health care personnel
- How would you define the within-practice allocation of roles between nurses, family physician and mental health specialist?
- Is it possible to conduct video consultations in a confidential treatment setting in your practice? If applicable, how can a confidential treatment setting be organized?
- How could the video consultations be scheduled in your practice?

*D. Specific parameters and necessary adaptions of the PROVIDE treatment model*

- Which models of financial remuneration do you consider appropriate?
- If applicable, what would you suggest to further optimize the treatment model?

*E. Interview termination*

- Do you have any further questions?
- Are there any remaining aspects important to you that we have not addressed at this point?
