# Supplementary material for: Perspectives of Psychotherapists and Psychiatrists on Mental Health Care Integration Within Primary Care Via Video Consultations: Qualitative Preimplementation Study
Source: J Med Internet Res. 2020 Jun 18;22(6):e17569. doi: 10.2196/17569 (PMC7333073; doi:10.2196/17569)
Supplement: Multimedia Appendix 3 [file jmir_v22i6e17569_app3.docx]

**Multimedia Appendix 3**

**Table MA3-1: Description of the coding system**

| *Key codes* | *Definition* | *Subcodes* | *Supporting quotes* |
| --- | --- | --- | --- |
| Individual health professional factors | Factors regarding attitudes and beliefs about the intervention model related to the individual mental health specialist | Outcome expectancy/potential benefit  (e.g. help for patients, collaboration with the family physician) | *“So for me it would be okay to do that without a personal added value. Because, I think, it may be more practical for the patients.”*  *[Participant 01, Focus Group 2]* |
|  |  | Barriers  (e.g. nonverbal communication, organizational) | *“Well, I have to say, I find that very difficult, yes. So I, I think having a personal counterpart, to speak to, is quite a very different situation.”*  *[Participant 02, focus group 1]* |
| Patient factors | Factors related to patients who might most likely benefit or not benefit from the model as well as factors promoting or inhibiting patient involvement in the intervention model | Target groups (e.g. patients with impaired mobility) | *“And to provide a supply or an offer for people who are just too far away, yes. Or when it is just not feasible to drive to the next city for a psychotherapy session.”*  *[Participant 03, focus group 3]* |
|  |  | Barriers (e.g. regarding technical aspects) | *Question: “Could you also imagine why a patient might refuse to participate?”*  *Participant: “Yes, being afraid of technology.”*  *[Participant 01, focus group 1]* |
| Professional interactions | Factors related to the cooperation and networking between the mental health specialist and the family physician. | Perceptions of responsibilities (e.g. mental health specialist as expert) | *“For the patient it is, so to say, a conversation with an expert.”*  *[Participant 03, focus group 3]* |
|  |  | Collaboration (e.g. brief case discussions) | *“So it would be very important that I talk to the family physician, with the colleague and not with any medical assistant.”*  *[Participant 01, focus group 3]* |
| *Key domains* | *Definition* | *Subdomains* | *Citation* |
| Incentives and resources | (Environmental) factors such as the availability of resources that promote the integration of the intervention into practice routines | Organizational resources (e.g. duration of the individual video consultation) | *“Organizationally, it would have to be sensibly integrable into everyday practice, somehow with stable network connectivity.”*  *[Participant 05, focus group 2]* |
|  |  | Financial incentives (e.g. for the individual video consultation) | *“So, assuming we had no costs for the infrastructure, I would confine myself with the normal payments.”*  *[Participant 01, focus group 3]* |
| Capacity for organizational change | System and context factors as well as readiness for change related to the practice routines | none (due to a small number of statements related to this domain) | *Question: “From your point of view, are mental health specialists willing to employ the proposed treatment model?”*  *Participant: “As with the patients, too. From open-minded to not at all open-minded.”*  *[Participant 03, focus group 2]* |
| Social, political and legal factors | Factors regarding social, political and legal prerequisites that may promote or hinder the integration of the intervention model into practice routines | n/a | n/a |
| Guideline factors | The extent to which the intervention model may be in line or conflicting with clinical practice guidelines | n/a | n/a |
